# Cyclic AMP Responsive Element Binding Proteins Are Involved in ‘Emergency’ Granulopoiesis through the Upregulation of CCAAT/Enhancer Binding Protein β

**DOI:** 10.1371/journal.pone.0054862

**Published:** 2013-01-30

**Authors:** Hideyo Hirai, Naoka Kamio, Gang Huang, Akiko Matsusue, Shinpei Ogino, Nobuhiko Kimura, Sakiko Satake, Eishi Ashihara, Jiro Imanishi, Daniel G. Tenen, Taira Maekawa

**Affiliations:** 1 Department of Transfusion Medicine and Cell Therapy, Kyoto University Hospital, Kyoto, Japan; 2 Division of Experimental Hematology and the Division of Pathology, Cincinnati, Ohio, United States of America; 3 Department of Clinical and Translational Physiology, Kyoto Pharmaceutical University, Kyoto, Japan; 4 Meiji University of Integrative Medicine, Kyoto, Japan; 5 Harvard Stem Cell Institute, Harvard Medical School, Boston, Massachusetts, United States of America; 6 Cancer Science Institute, National University of Singapore, Singapore, Singapore; Kanazawa University, Japan

## Abstract

In contrast to the definitive role of the transcription factor, CCAAT/Enhancer binding protein α (C/EBPα), in steady-state granulopoiesis, previous findings have suggested that granulopoiesis during emergency situations, such as infection, is dependent on C/EBPβ. In this study, a novel lentivirus-based reporter system was developed to elucidate the molecular switch required for C/EBPβ-dependency. The results demonstrated that two cyclic AMP responsive elements (CREs) in the proximal promoter region of C/EBPβ were involved in the positive regulation of C/EBPβ transcription during granulocyte-macrophage colony-stimulating factor (GM-CSF)–induced differentiation of bone marrow cells. In addition, the transcripts of CRE binding (CREB) family proteins were readily detected in hematopoietic stem/progenitor cells. CREB was upregulated, phosphorylated and bound to the CREs in response to GM-CSF stimulation. Retroviral transduction of a dominant negative CREB mutant reduced C/EBPβ mRNA levels and significantly impaired the proliferation/differentiation of granulocyte precursors, while a constitutively active form of CREB facilitated C/EBPβ transcription. These data suggest that CREB proteins are involved in the regulation of granulopoiesis via C/EBPβ upregulation.

## Introduction

Steady state granulopoiesis is a continuous process in which hematopoietic stem cells give rise to constant numbers of granulocytes, which are supplied to peripheral blood and various tissues [1]. During emergency situations such as infection, the supply of sufficient numbers of granulocytes to the front line is a prerequisite for the host defense. Under such a stressed condition, in addition to the mobilization of granulocytes from peripheral pools, granulopoiesis must be enhanced to meet immediate demands as granulocytes have a short half-life. Previous findings have suggested that, in steady-state conditions, granulopoiesis is dependent on the transcription factor CCAAT enhancer binding protein α (C/EBPα) [2], [3], whereas in emergency situations, granulopoiesis is dependent on C/EBPβ [4], [5]. Indeed, in response to cytokine stimulation or infection, the transcripts of C/EBPα were down-regulated in granulocyte precursors, while the transcripts of C/EBPβ were upregulated [4]. Both C/EBPα and C/EBPβ can induce granulocytic differentiation [4], [6], but the inhibitory effects of C/EBPα on cell cycle is much stronger than those of C/EBPβ [7], [8], [9]. This suggests a critical function for C/EBPβ in emergency granulopoiesis, which demands both differentiation and proliferation of granulocyte precursors. However, the molecular mechanisms involved in the shift from C/EBPα dependency to C/EBPβ dependency remain to be elucidated. In this study, a novel lentivirus vector-based reporter system was devised for monitoring the activity of a promoter of interest. This method was used to analyze the proximal promoter region of C/EBPβ and revealed the involvement of the cyclic AMP responsive element-binding (CREB) family of transcription factors in the regulation of emergency granulopoiesis through the transcriptional upregulation of C/EBPβ.

## Results

### Monitoring of Promoter Activity by a Lentivirus-based Reporter System

**Figure 1:**
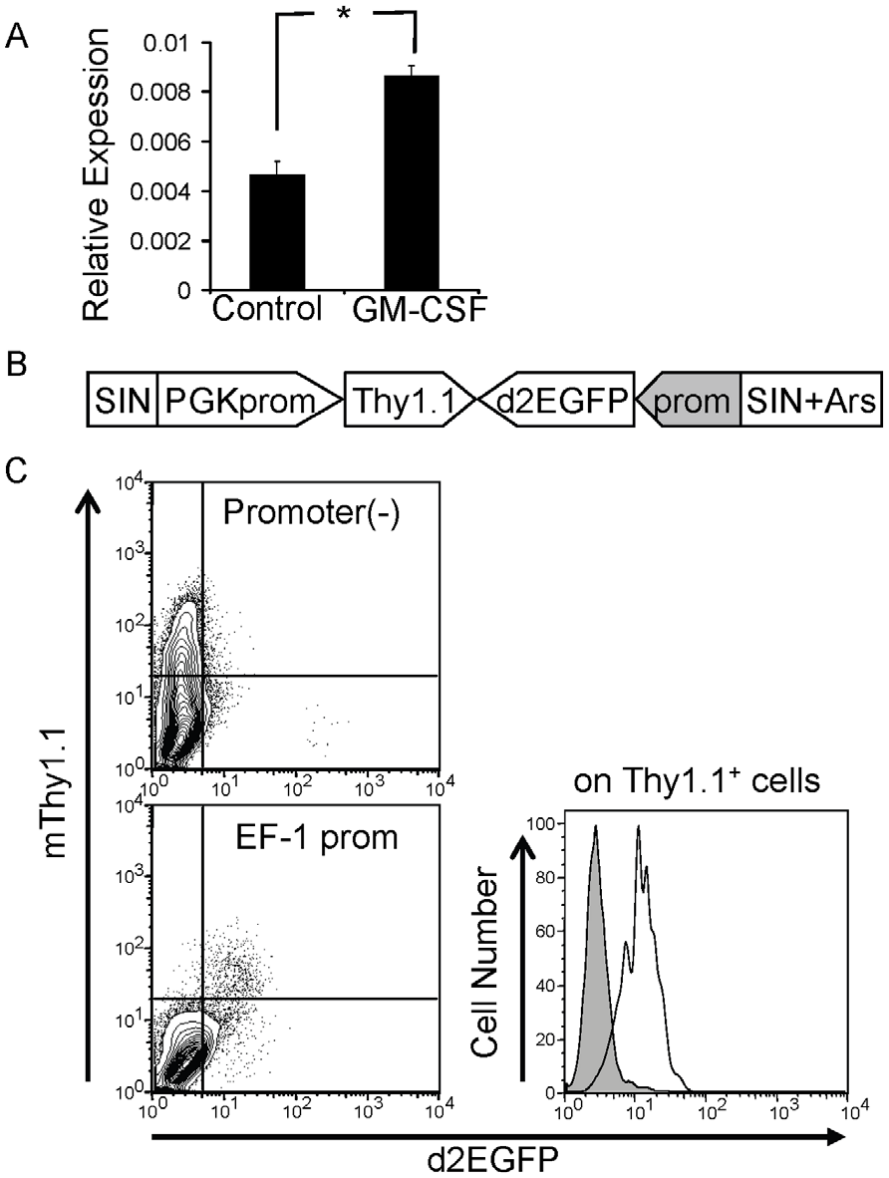
Lentivirus-based reporter system. A. Expression of C/EBPβ mRNA in c-kit^+^ bone marrow cells with or without GM-CSF stimulation *in vivo*. *:*P*<0.05, n = 3. Data are representative of two independent experiments. B. Schematic illustration of the lentivirus vector for reporter assays. SIN, self-inactivated long terminal repeat; PGK prom, phosphoglycerate kinase promoter; Ars I, sea urchin arylsulfatase insulator sequence. C. Elongation factor-1 (EF-1) promoter activity in bone marrow cells revealed by the lentivirus-based reporter system. Bone marrow cells were analyzed by flow cytometry after two days incubation with GM-CSF following viral infection. Shaded histogram, promoter-less control; open histogram, EF-1 promoter.

When GM-CSF was intraperitoneally administered to mice *in vivo* (10 µg/kg×2), C/EBPβ mRNA in c-kit^+^ bone marrow cells was upregulated approximately two-fold 48 hours after the injection (Figure 1A). This result, together with our previous observation [4], suggested that C/EBPβ is activated, at least partly, at the transcriptional level. The differentiation and proliferation of hematopoietic stem/progenitor cells are complicated processes, which are hard to mimic in cell lines; therefore, we established a system that allowed us to monitor the activity of a promoter of interest in primary cells. For this purpose, we took advantage of lentivirus vectors, which can be transduced into quiescent cells and easily applicable to human hematopoietic stem cells in the future. The two expression units shown in Figure 1B, a d2EGFP gene driven by a promoter of interest and a mouse Thy1.1 gene under the control of a constitutively-active PGK promoter, were cloned into a lentivirus vector. An insulator sequence from sea urchin arylsulfatase, Ars I [10], was cloned into the 3′ self-inactivated long terminal repeat of the vector to avoid integration site effects on promoter activity [11]. In this system, the activity of the promoter of interest will be reflected by the intensity of the d2EGFP signal within Thy1.1^+^ cells. To confirm the usefulness of this vector, bone marrow cells were transduced with the lentivirus vector with or without the elongation factor-1 (EF-1) promoter in front of the d2EGFP gene. EF-1 promoter activity was then assessed by flow cytometry 48 hours after the transduction (Figure 1C). As shown in the right panel, the expression level of d2EGFP within Thy1.1^+^ cells was enhanced strongly in the presence of the EF-1 promoter, confirming that this lentiviral system enabled us to monitor the activity of a promoter in mouse bone marrow cells.

### Location of a Positive Regulatory Element between −243 and −43 bp Upstream of the C/EBPβ Gene

**Figure 2:**
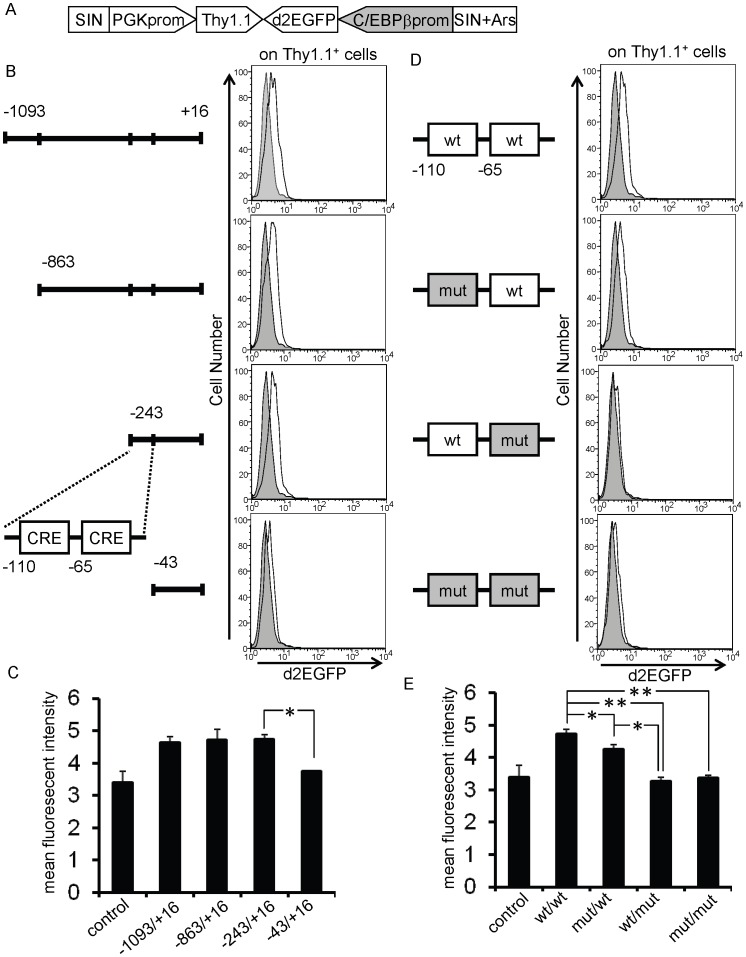
Flow cytometric analysis of the C/EBPβ proximal promoter region using the lentivirus-based reporter system. A. Schematic illustration of the lentivirus vector. SIN, self-inactivated long terminal repeat; PGK prom, phosphoglycerate kinase promoter; Ars I, sea urchin arylsulfatase insulator sequence. B and C. Bone marrow cells were transduced with the lentivirus vector containing the indicated fragment and were analyzed for Thy1.1 and d2EGFP expression after 48 hours stimulation with GM-CSF. D and E. Effects of the mutated CREs at −110 and −65 bp on the activity of the promoter fragment (−243 to +16 bp). Shaded histogram, promoter-less control; open histogram, promoter fragment. Wt, wild type; mut, mutated. *:*P*<0.05, **:*P<0.01* (n = 3). Data are representative of three independent experiments.

The proximal promoter region of the C/EBPβ gene was assessed using the lentiviral system mentioned above (Figure 2A). A 1106-bp genomic fragment (between −1093 and +16 bp) was cloned into the lentivirus vector and the promoter activity was investigated in GM-CSF–stimulated bone marrow cells. A shift in d2EGFP expression was observed in the presence of wild-type C/EBPβ promoter sequences (Figure 2B, top panel). Accordingly, the mean fluorescent intensity (MFI) was increased from 3.4±0.4 to 4.6±0.2 (p<0.05, n = 3, Figure 2C). When deletion variants of the genomic fragment were analyzed, the promoter activity was severely abrogated (MFI: from 4.7±0.1 to 3.7±0.1, p<0.05, n = 3), particularly when the fragment between −243 bp and −43 bp was deleted, suggesting the existence of positive regulatory elements within this region (Figure 2B, bottom panel and Figure 2C). A computational search for transcription factor consensus sequences within this region revealed two tandemly-aligned cyclic AMP responsive elements (CREs). To assess the function of these CREs with respect to the promoter activity, each CRE element (at −110 bp and −65 bp) was mutated using site-directed mutagenesis (Figure 2D). While the mutation in the CRE at −110 bp resulted in a slight but significant reduction of the promoter activity (MFI: from 4.7±0.1 to 4.3±0.1, p<0.05), the mutation in the CRE at −65 bp more efficiently impaired the promoter activity (MFI: from 4.7±0.1 to 3.3±0.1, p<0.01, n = 3, Figure 2D and E), suggesting that the CREs, especially the one at −65 bp, are important in mediating the response of the promoter to GM-CSF stimulation.

### CREBs in Myeloid Progenitors are Activated in Response to GM-CSF

**Figure 3:**
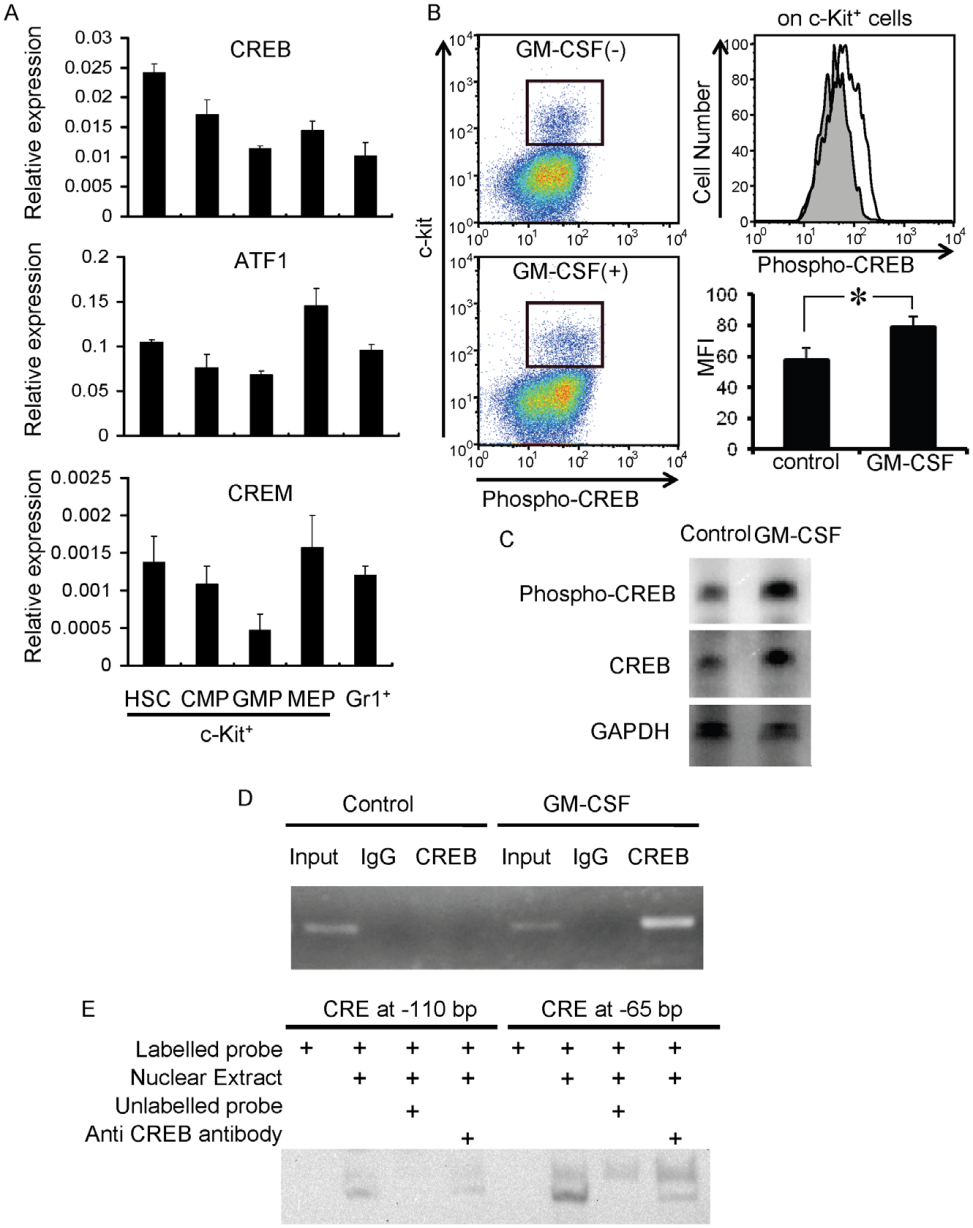
Expression and activation of CREB proteins in myeloid progenitors. A. CREB family member mRNA expression in purified hematopoietic populations. HSC, hematopoietic stem cells; CMP, common myeloid progenitor; GMP, granulocyte-macrophage progenitor; MEP, megakaryocyte-erythrocyte progenitor. B. Phosphorylation of CREB in response to GM-CSF. Left panels: expression of c-kit and phosphorylated CREB with or without GM-CSF stimulation. The expression of phosphorylated CREB within c-kit^+^ cells is shown in the right panel. Shaded histogram, without GM-CSF; open histogram, with GM-CSF. C. Western blotting analysis of c-kit+ bone marrow cells with or without stimulation with GM-CSF. D. Chromatin immunoprecipitation of CREB using bone marrow cells during GM-CSF–induced differentiation *in vitro*. E. Gel shift analysis with CREs in the promoter region of C/EBPβ and nuclear extracts of 293T cells transduced with constitutively active CREB. Data are representative of two independent experiments.

CRE is the consensus binding site for the CREB family of transcription factors which include CREB, ATF1 and CREM [12]. Firstly, the mRNA expression levels for these three proteins were measured in c-kit^+^ Sca-1^+^ lineage^−^ hematopoietic stem cells (HSCs), common myeloid progenitors (CMPs), granulocyte-macrophage progenitors (GMPs), megakaryocyte-erythrocyte progenitors (MEPs) and Gr-1^+^ mature granulocytes. Expression level of all three CREB family members were gradually decreased during the differentiation of HSCs to GMPs through CMPs, while the expression levels in mature granulocytes varied between the three factors (Figure 3A). MEPs expressed the transcripts of all these family members at higher level than GMPs (Figure 3A). These results are consistent with previous observations by Cheng JC *et al*. showing that CREB was highly expressed in immature hematopoietic cells [13]. CREB proteins are activated by the phosphorylation of specific residues, which leads to the recruitment of transcriptional coactivators and the activation of target gene transcription through binding to CREs [14], [15], [16]. When bone marrow cells were stained with a fluorescent-conjugated anti-phospho CREB-specific antibody, the intensity of the fluorescence within c-kit^+^ cells shifted after stimulation with GM-CSF (MFI: from 57.6±8.0 to 78.9±6.9, p<0.05, n = 3, Figure 3B), suggesting that phosphorylated CREB was induced by GM-CSF. Western blotting of c-kit^+^ cells before and after stimulation with GM-CSF showed the increase of both phosphorylated form and total CREB, suggesting that GM-CSF induced upregulation of overall amount of CREB (Figure 3C). Then, CREB protein binding to the CREs in the promoter region of C/EBPβ was assessed by chromatin immunoprecipitation using an anti-CREB antibody (Figure 3D). When a primer pair flanking both of the CREs was used to amplify the immunoprecipitated chromatin, the amount of the amplified product was enhanced in the presence of GM-CSF (Figure 3D). In addition, gel shift experiments were performed (Figure 3E). Nuclear extracts of 293T cells transduced with constitutively-active (CREB^DIEDML^) CREB [14] showed binding to both of the CREs. Addition of non-labeled oligonucleotide corresponding to CREB consensus sites abolished the binding and preincubation with anti-CREB antibody diminished the shifted bands. Taken together, these results suggested that the CREB proteins were expressed readily in hematopoietic stem/progenitor cells, upregulated and phosphorylated in response to GM-CSF, and bound to the CRE sites in the promoter region of C/EBPβ.

### Involvement of CREB Proteins in Cytokine-stimulated Granulopoiesis and C/EBPβ Upregulation

**Figure 4:**
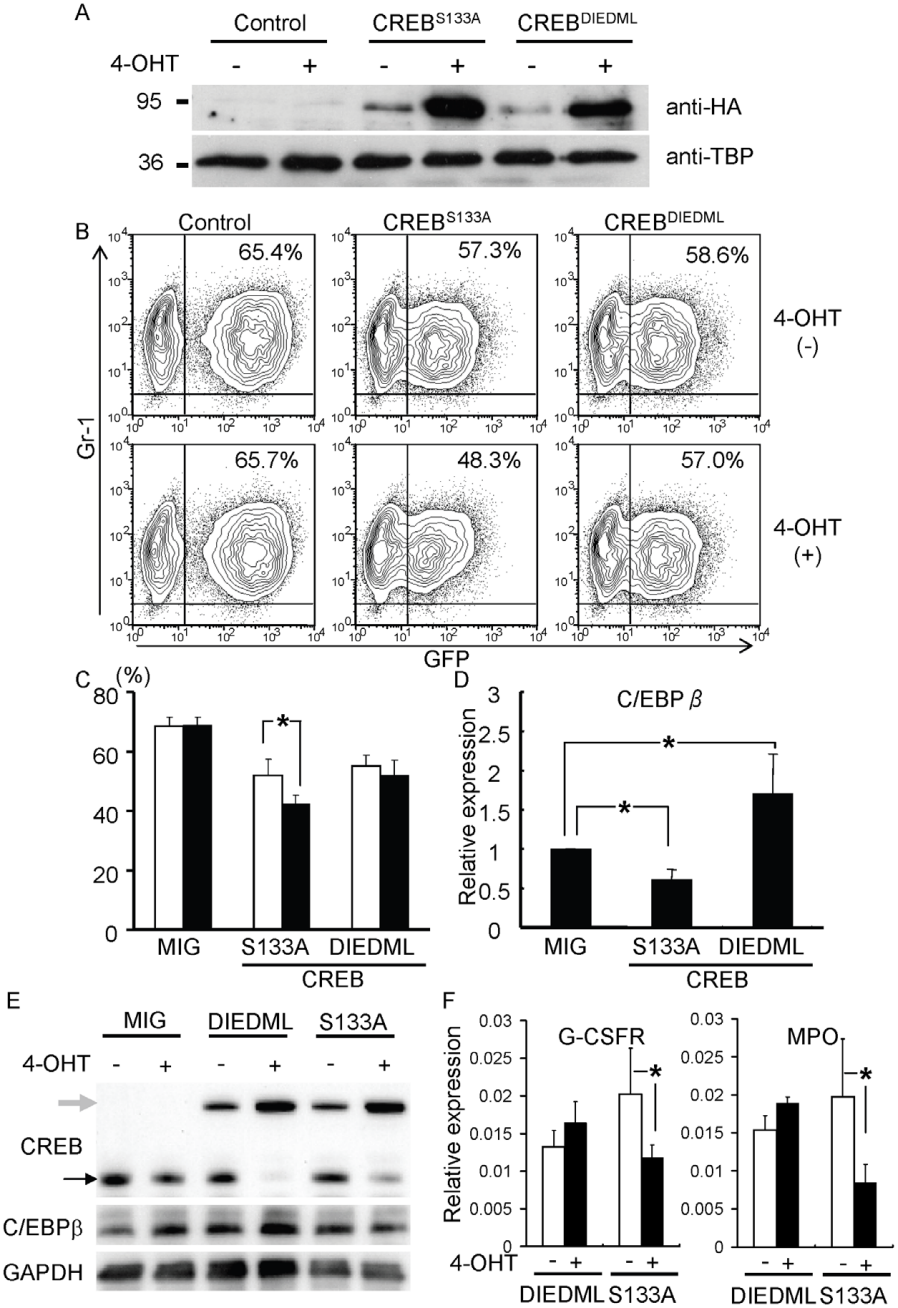
Effects of dominant negative and constitutively-active forms of CREB on GM-CSF–induced granulopoiesis. A. Western blotting of nuclear extracts from NIH3T3 cells retrovirally transduced with hemmagulutinin (HA) tagged-CREB mutants in the presence or absence of hydroxytamoxifen (4-OHT). TATA-binding protein (TBP) was used as an internal control of nuclear protein. B and C. Flow cytometric analysis of bone marrow cells stimulated with GM-CSF in the presence or absence of hydroxytamoxifen after transduction with the indicated vectors. The percentages of Gr-1^+^GFP^+^ cells for each experiment are shown in C. D. Expression of C/EBPβ mRNA in sorted Gr-1^+^GFP^+^ cells transduced with the indicated retrovirus vectors. *:*P*<005 (n = 3). E. Western blotting of whole cell lysates of GFP^+^ cells purified from bone marrow cells stimulated with GM-CSF in the presence or absence of hydroxytamoxifen after transduction with the indicated vectors. F. Expression of mRNA of G-CSF receptor (G-CSFR) and myeloperoxidase (MPO) in sorted Gr-1^+^GFP^+^ cells transduced with the indicated retrovirus vectors. *:*P*<005 (n = 3). Data are representative of two or three independent experiments.

To see the effects of CREB proteins on granulocytic differentiation and C/EBPβ mRNA expression, the dominant-negative (CREB^S133A^) [17] or constitutively-active (CREB^DIEDML^) [14] CREB mutants were fused to the ligand-binding domain of the estrogen receptor and were transduced into bone marrow cells from 5-fluorouracil treated mice using a bicistronic retroviral vector expressing GFP. Nuclear translocation of these fusion proteins in response to hydroxytamoxifen was confirmed by Western blotting of nuclear extracts from the transduced cell line (Figure 4A). The transduced bone marrow cells were cultured for a further 48 hours with GM-CSF in the presence or absence of hydroxytamoxifen. As shown in Figure 4B (middle panel) and 4C, treatment with hydroxytamoxifen significantly reduced the GFP^+^ Gr-1^+^ cells when the dominant negative CREB mutant (CREB^S133A^) was transduced, whereas it had no significant effect following the transduction of the control vector or a vector expressing the constitutively active CREB^DIEDML^ (Figure 4B, left and right panels, respectively). The GFP^+^Gr-1^+^ cells from these experiments were then subjected to quantitative RT-PCR. The introduction of the CREB^S133A^ mutant repressed C/EBPβ mRNA expression significantly, whereas CREB^DIEDML^ upregulated C/EBPβ by almost two-fold (p<0.05) (Figure 4D, left panel). Nuclear translocation of either CREB^DIEDML^ or CREB^S133A^ downregulated the endogenous CREB protein and resulted in upregulation or downregulation of C/EBPβ protein, respectively (Figure 4E). Then we measured the expression levels of putative targets of C/EBPβ. CREB^S133A^ mutant significantly downregulated the transcripts of G-CSF receptor and myeloperoxidase. In contrast, CREB^DIEDML^ did not affect the expression of these genes. These results suggested that CREBs are involved in the cytokine-stimulated granulopoiesis through the upregulation of C/EBPβ.

**Figure 5:**
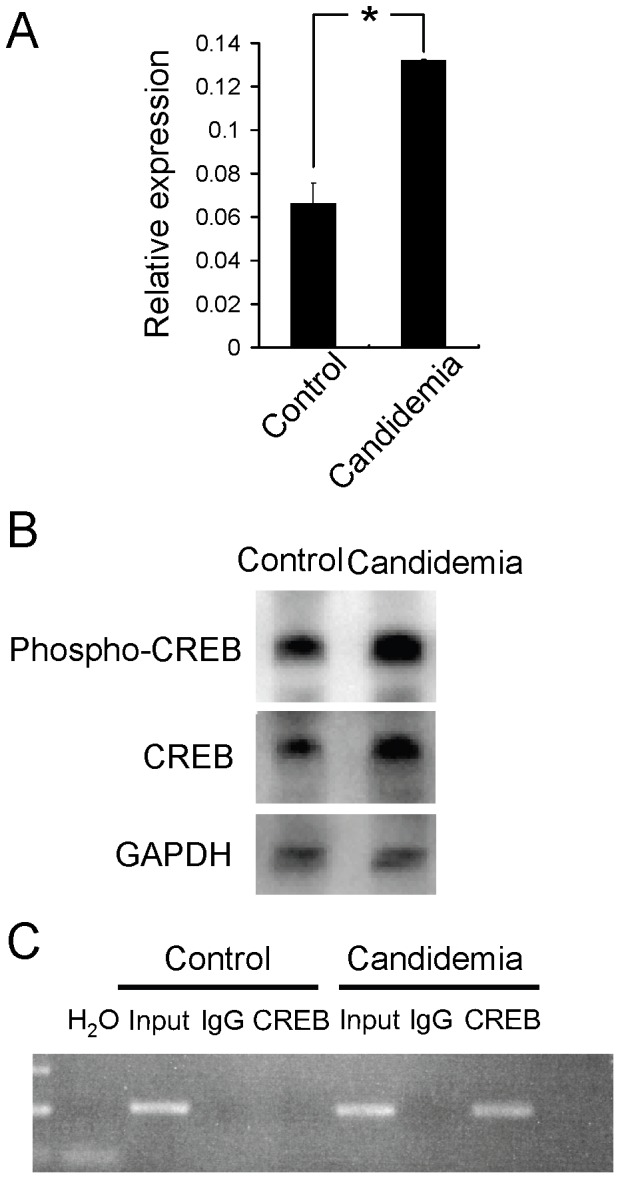
Involvement of CREB-C/EBPβ pathway in candidemia-induced “emergency granulopoiesis.” A. Expression of C/EBPβ mRNA in c-kit^+^ bone marrow cells with or without candidemia. *:*P*<0.05, n = 3. B. Western blotting analysis of c-kit+ bone marrow cells with or without candidemia. C. Chromatin immunoprecipitation of CREB using c-kit+ bone marrow cells during candidemia induced emergency granulopoiesis. Data are representative of two independent experiments.

In order to verify that CREB mediated upregulation of C/EBPβ is involved in emergency granulopoiesis, a fungus infection experiment was carried out. Tail vein injection of *Candida albicans* causes enhanced granulopoiesis [4], [18], [19]. c-kit^+^ cells were purified from bone marrow harvested on day 2 after infection and subjected to quantitative RT-PCR. C/EBPβ mRNA was upregulated approximately two-fold (Figure 5A). CREB was upregulated at protein level and binding of CREB to the promoter region of C/EBPβ containing the CREs was also enhanced as revealed by chromatin immunoprecipitation experiments (Figure 5B and 5C), suggesting that CREB-C/EBPβ pathway is activated in candidemia-induced emergency granulopoiesis.

## Discussion

The activation of C/EBPβ is controlled at multiple steps, from transcription and translation through to post-translational modifications, in response to various different stimuli [20]. This study has focused on the analysis of C/EBPβ transcriptional regulation during GM-CSF–induced granulocytic differentiation using a lentivirus-based reporter assay system developed for the purpose, and the results clearly demonstrated that members of the CREB family of transcription factors are involved in regulating the transcription of C/EBPβ during cytokine-stimulated granulopoiesis.

Sakamoto and colleagues have investigated the role of CREB in hematopoiesis intensively. They showed that CREB levels are elevated in normal immature bone marrow cells and blast cells from patients with acute myeloid leukemia [13], [21]. Transgenic mouse lines expressing CREB under the control of the myeloid specific MRP8 promoter developed myeloproliferative syndrome with splenomegaly [21], while the knockdown of CREB using siRNAs resulted in the decreased proliferation of normal and leukemic hematopoietic stem/progenitor cells [13]. Pigazzi et al. also have shown that dysregulation of CREB was involved in the pathogenesis of leukemia [22], [23]. These results clearly suggest that CREB proteins play important roles in the proliferation and differentiation of normal and malignant myeloid cells. In this study, a dominant negative mutant of CREB downregulated the expression level of C/EBPβ and decreased the Gr-1^+^ granulocytes (Figure 4). These results suggested that CREBs are also involved in the cytokine-stimulated granulopoiesis and that C/EBPβ is one of the downstream targets of CREB. When a constitutively active CREB was transduced, increase of Gr-1^+^ granulocytes was not observed in spite of the upregulation of C/EBPβ (Figure 4B, 4C and 4D). In the presence of GM-CSF, C/EBPβ might be already fully activated for induction of granulocytic differentiation. Therefore, further upregulation of C/EBPβ by extrinsically introduced constitutively active CREB failed to cause further increase of Gr-1^+^ cells.

The connection between CREB proteins and C/EBPβ has also been observed in other tissues. In hepatocytes, CREB proteins regulate C/EBPβ transcription through the CREs in response to lipopolysaccharides (LPS) stimulation [24] or during liver regeneration after partial hepatectomy [25]. During the mitotic clonal expansion of adipocytes, a process of early adipocyte differentiation, C/EBPβ is also under the control of CREB [26]. In hematopoietic systems, C/EBPβ is transcriptionally upregulated in monocytes and macrophages when they are activated by LPS [27], [28]. Here, we showed the involvement of CREB-C/EBPβ connection in granulopoiesis for the first time.

There are two classes of CREs, asymmetric and weak binding sites and symmetrical sites with higher binding affinity. Constitutive binding of CREBs is observed in the symmetrical CREs and phosphorylation-dependent binding is specific to the asymmetric and weak binding sites [29]. Both of the CREs in the C/EBPβ promoter are the asymmetric and weak binding sites. The CRE sites in the promoter region of C/EBPβ are conserved from *Xenopus* to human [25], [27], [30], [31], suggesting that the transcriptional regulation of C/EBPβ by CREB is a fundamental biological process required for the maintenance of homeostasis under various stress conditions.

In patients with congenital neutropenia (CN), steady state granulopoiesis driven by lymphoid enhancer-binding factor (LEF-1) and C/EBPα is almost abrogated [32], and treatment of these patients with G-CSF triggered C/EBPβ-dependent emergency granulopoiesis, which resembled the C/EBPα-independent and C/EBPβ-dependent ‘emergency’ granulopoiesis previously observed in a mouse model. [4] In a subset of leukemia cells, impairment of the function or expression of C/EBPα results in a differentiation block in leukemic blast cells [33]–[35], which might possibly be reversed by the upregulation of C/EBPβ [36]. Here, CREBs-mediated transcriptional activation of C/EBPβ was shown as one of the switching mechanism for ‘emergency’ granulopoiesis. Recent reports have demonstrated that STAT3 and sirtuin-1 were also involved in the upregulation of C/EBPβ during granulopoiesis under stressed conditions [37]–[38]. Further elucidation of the molecular mechanisms regulating C/EBPβ will facilitate the improvement of therapies for treating patients with diseases in which C/EBPα is dysregulated, such as CN and leukemia.

In this study, we showed that CREB proteins are involved in the regulation of granulopoiesis through the transcriptional upregulation of C/EBPβ, suggesting that CREB is a molecular switch required for C/EPBβ-dependent granulopoiesis. In addition, the novel lentivirus vector-based reporter system will facilitate the analysis of gene regulatory elements in primary cells.

## Materials and Methods

### Ethics Statement

All procedures using animals were reviewed and approved by the Kyoto Prefectural University of Medicine Animal Research Committee (Permit Number: M18-55).

### Mice

C57BL/6 mice were purchased from Shimizu Laboratory Animals (Kyoto, Japan) or Clea Japan (Tokyo, Japan). We took all necessary steps to minimize suffering of mice during the experiments.

### Infection with Candida Albicans

Systemic infection with *Candida albicans* were carried out as previously described [4], [18], [19]. Briefly, *Candida albicans* (18804; American Type Culture Collection) was plated onto Luria-Bertani broth agar plates and the plates were stored at 4°C for a maximum of 4 weeks. Before each experiment, several colonies were picked from the plate and grown in 3 mL of Sabouraud dextrose broth (Sigma Aldrich, St. Louis, MO) at 37°C for 24 h. The fungi were washed twice with pyrogen-free phosphate-buffered saline (PBS), re-suspended in PBS, and 4×10^6^ CFU/20 g body weight/mouse were intravenously injected *via* the tail vein to induce disseminated candidiasis.

### Cell Lines

NIH3T3 cells (ATCC) were cultured in Dulbecco’s modified Eagle’s medium (Wako, Japan) supplemented with 10% fetal bovine serum at 37°C in 5% CO_2_.

### C/EBPβ Promoter

A 1109-bp region from the proximal promoter (−1093 bp to +16 bp; transcription start site: +1 bp) of C/EBPβ was amplified by PCR using the following genomic DNA primers: forward, cggaattcacgcgtggttgagcaacaccccaccagcttgccggggc; reverse, ccgctcgaggtcccgtgcgcggctcggactcggctcggcgg. The PCR product was cloned into the *EcoR*I and *Xho*I sites of a pBluescript plasmid vector. A series of deletion variants of the promoter (−863/+16 bp, −243/+16 bp and −43/+16 bp) were generated by removing the fragments between *Aat*I/*BstE*II, *Aat*I/*Nhe*I or *Aat*I/*Nar*I, respectively, and allowing the vector to religate. Mutations that disrupt the CRE sites at −110 bp and −65 bp of the promoter were generated by PCR-based, site-directed mutagenesis as previously described [39] and changed the sequence at each site from TGACG to ACTCG. [25]. The introduced mutations and the integrity of non-mutated regions were confirmed by sequencing of cloned PCR products.

### Lentivirus Vectors

Two expression units, i) a destabilized variant of the enhanced green fluorescent protein (d2EGFP derived from the pd2EGFP vector, Clontech, CA), and ii) the mouse *Thy1.1* gene under the control of the phosphoglycerate kinase (PGK) promoter, were flanked by an *Mlu*I-*EcoR*I CS-CDF-EG-PRE fragment (Riken BioResource Center) and an *Xba*I-*Mlu*I CS-II-Ars(-) fragment [11] to form the lentivirus vector, CS-PGKThy-d2G-MCS. The various C/EBPβ promoter sequences between *EcoR*I and *Xho*I in pBluescript were cloned into the multi cloning site of CS-PGKThy-d2G-MCS.

### CREB Mutants

Plasmid vectors containing CREB^S133A^ (pNN LBDCREB^S133A^) [17], a dominant negative CREB mutant, and CREB^DIEDML^ (pRC/RSV FLAG CREB^DIEDML^) [14], a constitutively-active CREB mutant, were kind gifts from Dr. S. Kida and Dr. R. H. Goodman, respectively. These mutants were fused to the ligand-binding domain of the estrogen receptor and hemagglutinin virus (HA)-tag sequence and cloned into the MSCV-IRES-GFP vector.

### Lentivirus and Retrovirus Infection

Viral supernatants were collected as previously described [40] (see also http://www.brc.riken.jp/lab/cfm/Subteam_for_Manipulation_of_Cell_Fate/Protocols.html). Bone marrow cells were harvested from 5-fluorouracil–treated mice and cultured in the presence of stem cell factor (10 ng/ml), interleukin (IL)-3 (10 ng/ml) and IL-6 (10 ng/ml) for two days. Viral infection was carried out twice during the following two days with 4 µg/ml of polybrene. Cells were analyzed by flow cytometry after a further two days culture in the presence of granulocyte-macrophage-colony–stimulating factor (GM-CSF) (10 ng/ml, R&D systems, MN). Transduction efficiency was around 10% (4–30%) and was dependent on the length of the promoter inserted in the vectors and on the transduction condition.

### Detection of Phosphorylated CREB by Flow Cytometry

After 1 hour of stimulation with GM-CSF, bone marrow cells were fixed with 20 volumes of prewarmed BD Phosflow Lyse/Fix buffer (BD Bioscience, CA), incubated at 37°C for 10 minutes, permeabilized with BD Phosflow Perm III (BD Bioscience, CA), and incubated on ice for 30 minutes. Permeabilized cells were then resuspended in BD Pharmingen stain buffer with FcBlock (BD Bioscience, CA). Then, cells were stained with allophycocyanin (APC)-conjugated anti c-kit antibody and Alexa Fluor® 488-conjugated anti phospho-CREB antibody (Cell Signaling Technology, MA).

### Flow Cytometric Analysis

Mouse bone marrow cells were analyzed and sorted using a FACSCalibur or FACSAria flow cytometer (BD Bioscience, CA). Data were analyzed with FlowJo software (Tree Star Inc., OR).

### Quantitative RT-PCR

Total RNA was extracted using the RNeasy Micro kit (Qiagen, CA) and reverse-transcribed using random primers. The real-time PCR was performed as previously described [41]. The real-time PCR reaction mixture contained 4µl of Taqman master mix (Roche Diagnostics GmbH Mannheim, Germany), cDNA, pairs of primers and Taqman probe (Universal Probe Library, Roche Diagnostics). The cDNA was amplified with a Light Cycler 3.5 (Roche Diagnostics) using the following parameters, 95°C for 10 minutes, followed by 45 cycles at 95°C for 10 seconds and 60°C for 30 seconds. Glyceraldehyde-3-phosphate (GAPDH) was used as an internal control with nuclease-free water as a negative control. The sequences of the primers and probes are as follows: CREB, forward primer: ccaaactagcagtgggcagt, reverse primer: ccccatccgtaccattgtt, Roche Universal Probe #52; activating transcription factor 1 (ATF1), forward primer: agagaaatacgactgatgaaaaacag, reverse primer: ttttcagaacagcaacacg, probe #80; cyclic AMP responsive modulator (CREM), forward primer: gctgaggctgatgaaaaaca, reverse primer: gccacacgattttcaagaca, probe #4; C/EBPβ, forward primer atcgacttcagcccctacct, reverse primer: tagtcgtcggcgaagagg, probe #55; C/EBPα, forward primer ccttcaacgacgagttcctg, reverse primer: tggccttctcctgctgtc, probe #11; and GAPDH, forward primer: tgtccgtcgtggatctgac, reverse primer: cctgcttcaccaccttcttg, probe #80.

### Chromatin Immunoprecipitation

Chromatin immunoprecipitation experiments were performed using a Chip IT-Express kit according to the manufacturer’s instructions (Active Motif, Rixensart, Belgium). For immunoprecipitation of the fragmented chromatin, an anti-CREB antibody (48H2, Cell Signaling Technologies, MA) was used. The following set of primers were used for the amplification of the genomic fragment containing the two CREs in the proximal promoter region of C/EBPβ, forward: tcacccgcgtccgtg, reverse: gccgagcgggaggttta.

### Western Blotting

Cells were diluted with Laemmli sample buffer and boiled at 100°C for 10 min. Samples were separated by SDS-PAGE and transferred to polyvinylidene fluoride membranes. Antibodies specific for C/EBPβ (sc-150, Santa Cruz Biotechnology, Santa Cruz, CA, USA), CREB (sc-25785, Santa Cruz Biotechnology), TBP (sc204, Santa Cruz Biotechnology), HA (sc-805, Santa Cruz Biotechnology) and GAPDH (sc-25778, Santa Cruz Biotechnology) were used as primary antibodies. Immunoreactive proteins were detected using horseradish peroxidase-conjugated anti-rabbit IgG (NA934V, GE Healthcare, Little Chalfont, UK) and visualized using enhanced chemiluminescence (ECL, GE Healthcare).

### Gel Shift Assay

Nuclear ectracts from transduced 293T cells were isolated with NE-PER™ kit according to the manufacturer’s instructions (Pierce, Rockland, IL) and the LightShift Chemiluminescent Electrophoretic Mobility Shift Assay Kit was used for detection of DNA-protein interaction. CRE sites oligonuculeotides contained the following sequences: CRE at −110 bp, 5′-GGGCAATGACGCGCACCGACCG-3′; CRE at −65 bp, 5′-CCAGCGTGACGCAGCCCGTTGC-3′; consensus CREB binding sites, 5′-AGAGATTGCCTGACGTCAGAGAGCTAG-3′. Binding reaction was performed on ice for 20 min (0.2 µM biotin-labeled oligonucleotide probe, 1×binding buffer, 150 µmol/L KCl, 0.1 µmol/L EDTA, 2.5 µmol/L DTT, 0.05% NP40, 10% glycerol, and 50 ng/mL poly[dI-dC]).

### Statistical Analysis

Statistical differences were determined by the Student’s *t* test. *P* values <0.05 were considered statistically significant.
